# Exploring the Multidisciplinary Factors Affecting Sports Talent Identification

**DOI:** 10.3389/fpsyg.2022.948121

**Published:** 2022-07-11

**Authors:** Changqing Xiang, Tengku Fadilah Tengku Kamalden, Hejian Liu, Normala Ismail

**Affiliations:** ^1^Department of Sport Studies, Faculty of Educational Studies, Universiti Putra Malaysia, Serdang, Malaysia; ^2^Office of Scientific Research, Guangzhou University, Guangzhou, China; ^3^School of Education, Guangzhou University, Guangzhou, China; ^4^Department of Science and Technical Education, Faculty of Educational Studies, Universiti Putra Malaysia, Serdang, Malaysia

**Keywords:** sport talent, factors, physical education, identification, SEM

## Abstract

Talent is one of the most significant factors to promote the development of sports undertakings. The present study aimed to explore the factors affecting the identification of sports talents in China's physical education curriculum. Based on the literature review, this study puts forward a model to examine the influencing factors of sports talent identification in China's physical education curriculum using structural equation modeling and uses the structural equation modeling and factor analysis method to verify the hypothesis combined with the results of 310 effective questionnaires. The article summarizes influencing factors from four aspects, namely, physical, psychological, coach, and environmental factors. On the basis of relevant literature, the hypothesis model was established by structural equation modeling. The results show that the main factors affecting the identification of sports talents in the physical education curriculum are personal physical quality performance, psychological quality, coach's knowledge, and the identification policies of schools to sports talents. The conclusion of this study can provide guidance for the reform of the physical education curriculum, the growth of sports talents, and the development of sports talents in China.

## Introduction

The sports talent identification (TID) refers to the discovery of potential athletes in a heterogeneous population that are currently not involved in a specific sport (Vaeyens et al., 2008). TID is a key area within sports development, and TID is an inseparable from the growth of athletes. As a key part of cultivating sports talents, the physical education (PE) curriculum plays a significant role in transporting sports reserve forces for national sports. At the same time, TID could be a helpful tool to stimulate lifelong sports participation and reduce dropouts because it can reveal an optimal connection between sports, individual strengths, and personal preferences (Pion et al., 2015). Meanwhile, there are some differences talent detection, identification, development, and selection. Talent detection is the first stage involved in the conversion of a talented player into an elite sportsman (Ricard et al., 2018), and talent detection intends to support lifelong sports participation, reduce dropouts, and stimulate sports at the elite level (Faber et al., 2017). TID is predicting students or players who have the potential skills to development into elite athletes (Williams and Reilly, 2000); talent development processes involve experienced, well-qualified coaches' careful training, academic education (Ford et al., 2020), athletic rehabilitation, athletic load capacity control, and professional sports skills guidance to athletes; and talent selection mainly concerns about choosing the most appropriate (group of) athletes to complete a specific task (in a team) in sports (Williams and Reilly, 2000). In short, talent detection, identification, development, and selection compose the whole process that runs through different stages of sports talent, and each their own characteristics and requirements.

Although China is a big sports country, there is still a long way to go between China and a powerful sports country. In October 2020, the Chinese government issued the “Opinions on Comprehensively Strengthening and Improving School Physical Education in the New Era,” which put forward that school PE, as a basic project to improve students' all-sided quality, is vital to accelerate the modernization of education and build a powerful country in education and sports, and on this account establish and improve the sports competition system and talent training system, in order to identifying and cultivating more excellent sports reserve talents.

School PE should gradually strengthen the selectivity and hierarchy, further enhance students' interest and behavior in participating in sports, and pay more attention to lifelong PE for students; competitive sports should be popular again in school PE, and the detection of sports talents should be an indispensable part of the school PE curriculum. Bailey and Morley (2006) have presented a model of talent in PE, indicating that excellent school PE helps implement effective talent development plan, and clarified the purpose and value of PE.

At the same time, sports talents are greatly restricted by age characteristics, and the improvement of sports performance can be made within the appropriate age range (Haycraft et al., 2018); therefore, sports talent needs to be identified at early stages so that they can be nurtured to develop elite athletes. Therefore, the aim of the present study was to explore the factors that affect the TID in the school PE curriculum.

### Sport TID Research

The notion of talent and its development have been explored in other domains, and while providing some intriguing and relevant elements for those working in sport settings, these domains may be insufficient to capture the complexity of talent in sports (Joseph et al., 2019). Many scholars have carried out much relevant research on TID before including multidisciplinary, longitudinal, prospective, or qualitative studies, such as the investigation and research on the process of sports professional TID (Ford et al., 2020), the current model and future development direction of TID in sports (Vaeyens et al., 2008), and the predictive factors in the process of TID (Williams et al., 2020). In terms of the concept and content of sport talent, it is to point to a career in sports, sports professional knowledge, skills, and technical talents, mainly includes the athletes, coaches, sports scientific research personnel, sports management, sports teacher, entrepreneur, and sports agent for the development of undertakings of physical culture and sports as well as promote social progress, has made certain contribution or have some contribution to the potential of people. TID is a multi-factorial process (Ricard et al., 2018) and relate to identifying people who participate in sports with the potential to become professional athletes through training and cultivating.

According to the theory of subject knowledge, a multidisciplinary approach is used for TID including physical, technical, and psychological predictor variables in players (e.g., adult height, maturity, sprinting, dribbling skills, and intrinsic motivation) (Williams and Reilly, 2000; Ford et al., 2020). Hence, TID is a linear process, where sports competitions source athletes for the existing vacancies. This linear process is reactive in its nature and gift, thus lead to increased time to finding and cost to cultivating. From of the aforementioned discussion, we can know that there is increasing research on sport TID carried out around the concept, method, content of sport TID, and other aspects related to TID. As an important part of cultivating sports talents, PE class plays a significant role in transporting sports reserve forces for national sports, as well as sport TID.

### Influencing Factors of Sport TID

In this process, TID is associated with many aspects including not only anthropology, pedagogy, physiology, and psychology but also sociology and coaching science (Williams and Drust, 2012). In another words, TID is an important stage of talent growth, and in this complex and multidimensional process and is affected by multi-dimensional factors. There have been many research studies on TID in the past decade, including methods of TID (Vladan et al., 2009; Clarke et al., 2018; Kyle et al., 2019; Williams et al., 2020) and identification factors (Norikazu and Taigo, 2016; Gledhill et al., 2017; Dodd and Newans, 2018; Lai and Ishizaka, 2020). Among them, physical factors are more discussed in TID. Genetic factors are considered to play a critical role in athletic performance and related phenotypes (Miah and Rich, 2006; Ahmetov et al., 2016; David et al., 2017). There is a consensus in the scientific and sporting communities that genetic factors contribute to athletic performance. According to research, at least 155 genetic markers were found to be related to elite athlete performance (93 genetic markers related to endurance and 62 genetic markers related to power/strength). Meanwhile, genome-wide association studies (GWASs) are the most commonly used method to identify athletic performance in athletes (Ahmetov et al., 2016). Components like height, body fat percentage, size of palm, dynamic balance, static balance, and hand strength were the key elements in TID of badminton (Mojtaba et al., 2012). Therefore, anthropometric factors index plays a crucial role in TID process.

Psychological factors are also very significant in the process of TID and talent development, and a focus that has grown markedly over the last decade or more. Saby et al. (2020) used a longitudinal method to examine changes in appraisals, emotion regulation, and emotions in adolescent soccer players based at a competitive season in France and found intertwined psychological constructs in a dynamic relationship allowing athletes to continuously adjust to their constantly changing everyday demands. Similarly, Saward et al. (2016) used a mixed-longitudinal prospective method to examine the psychological characteristics associated with elite youth soccer players aged 8–18 years in three seasons. Apart from these, Rongen et al. (2020) used a prospective longitudinal cohort design to track psychosocial outcomes of academy involvement in male youth elite soccer players and suggested that negative psychosocial impacts of soccer academy involvement did not materialize in this context. Enrique and William (2006) through 24 in-depth semi-structured interviews with adolescents who involved in competitive sports concluded that motivational roles are related to the interpersonal influence on adolescents' sports motivation.

Compared with the first two factors, sociological factors are less discussed, but there are still some research on this. For instance, self-regulation and adaptive volitional behaviors appear to be key intra-individual factors associated with talent development (Haugaasen et al., 2014), and a number of talent development environmental factors (e.g., media coverage, sports participation rate, birthplace, long-term development, and quality preparation) also exist in sports (Li et al., 2014). On the whole, according to the current research, there are many factors affecting the sport TID, mainly physiological, psychological, and sociological factors, which play a vital role in both the identify of sports talents and the development of sports talents.

### Current Study

Although there are a great deal of studies on the influencing factors of sport TID, most of them focus on a specific aspect, such as the relative age effect (RAE), athletes' psychological characteristics, and students' growing environment around these factors. This study used the relevant literature to analyze the multidisciplinary factors affecting sport TID as much as possible and verified these influencing factors through structural equation modeling as a way to better improve the success rate of sports talent. In this study, on the basis of a questionnaire survey, a Likert scale was used to assign scores to the listed influencing factors, and the data collected by questionnaire were analyzed. The questionnaires were distributed to PE teachers, sports administrators, and coaches, who were asked to rate the degree of influence of the factors in the questionnaire. The analysis of the data shows that physical, psychological, coach, environmental factors all have a positive impact on the sport TID.

As an integral part of education, PE is a purposeful, planned, and organized educational process conducted through physical activities and other auxiliary means (Mountakis, 2001). In a systematic review, Prieto-Ayuso et al. (2020) showed that methods and instruments for talent identification in PE are changing, so it is important to select appropriate programs to deal with gifted children. In addition, PE teachers in the development of the scale can help speed up the identification of potential talents (Platvoet et al., 2015); however, teachers in school PE still face certain difficulties within the identification process (Bailey et al., 2009).

In another aspect, there are many factors influencing sport TID in the school PE curriculum, due to the complex environment and existing regulations. Thus, based on the literature, expert consultation, and the factors associated with TID, a questionnaire was designed and distributed through online and offline. Researchers should have a reasonable theoretical basis and specific literature support when setting up models (Hoyle and Panter, 1995; Boomsma, 2000; McDonald and Ho, 2002). The setting of the model in this study includes relevant theories of the variables in the research model and the essence of past research. In this research, the authors selected physical, psychological, coach, and environment as four external variables, corresponding to 12 observation variables, respectively, and sport TID is as internal latent variables.

Although the existing literature on sport TID is very extensive, there are still some deficiencies. The development of school PE promotes the sport TID, and the development of sport TID will promote the innovation and progress of the school PE curriculum. However, the sport TID in the school PE curriculum is affected by many factors. For this reason, identifying the factors that affect TID in the school PE curriculum can improve the identification rate of school sports talent and further consolidate the strength of sports reserve talent.

This research aims to reveal factors influencing sport TID in the PE curriculum in China, and the following questions will be discussed in this research:

(1) What is the current situation of sport TID and how to classify the factors influencing sport TID in the school PE curriculum in China?
(2) What are the specific factors that affect TID in the school PE curriculum in China?
(3) How to make the strategy of TID in the school PE curriculum according to the situation of school PE in China?

### Hypothesis

Physical quality is not only the foundation guarantee of athletes but also the basis of special sports quality. On the one hand, Norikazu and Taigo (2016) through 2-year follow-up measurements of soccer players found that factors of the anthropometric index such as sprint ability is a useful identification index and muscular power is limited and useful in TID of soccer. On the other hand, physiological aspects included anthropometrical, linear speed, change of direction speed (CODS), maximal anaerobic power, repeated sprint ability (RSA), maximal aerobic power, and maximal lower body strength, and they should be taken into account in the TID of soccer in the testing batteries (Dodd and Newans, 2018). Hence, we propose the following hypotheses:

(1) H1: Physical quality has an influencing on sport TID.
(2) H1a: Height and weight positively affect sport TID.
(3) H1b: Motor ability index advantage positively affects sport TID.
(4) H1c: The quality of the anthropometric index positively effects sport TID.

Psychological influence sport TID means the mentality maturity of the personal are advantaged on performance tests (Vaeyens et al., 2008), psychological factors manifesting itself at various levels, having a predominantly unconscious nature. Likewise, a systematic review also indicated that psychological factors (e.g., adaptive perfectionism, task/mastery orientation, delaying gratification, and coping strategies) are associated with talent development in football and suggested that psychological characteristics of self-regulation, resilience, commitment, and discipline appear to be most impactful on TID (Gledhill et al., 2017). Based on this, this research propose the following hypotheses:

(1) H2: Psychological quality has an impact on sport TID.
(2) H2a: Sports motivation significant affects sport TID.
(3) H2b: Personal qualities positively affect sport TID.
(4) H2c: Students' cognition of sports positively affects sport TID.

In the same way, coaches' knowledge is associated with talent detection, but it is unable to articulate how or in what ways they see talent (Roberts et al., 2019). An increasing number of researchers have explored the value of the “coach's eye” in TID and talent evaluation settings. For instance, a research study through systematic review and meta-analysis describing a model from decision-making concluded that a coach's eye is subjective and experience-based, and these criteria can differ from coach to coach (Lath et al., 2021). In another aspect, in the process of TID, because the coach's eye is based on intuition, coaches often fail to state clear variables and reasons for their selection decisions (Roberts et al., 2019). Therefore, we propose the following hypotheses:

(1) H3: The coach's knowledge has an impact on sport TID.
(2) H3a: The professional level positively affects sport TID.
(3) H3b: Relationship with students positively affects sport TID.
(4) H3c: Management ability positively affects sport TID.

Social and environmental factors that can impact upon TID (Mills et al., 2014): John et al. (2019) through systematic review found that critical life events have an impact on developmental pathways of elite athletes. In the meantime, from another point of view, the organization operation and the human resource management (HRM) system are commonly associated with talent management, whereas talent development can be further enhanced by influencing personal and environmental catalysts (Meyers et al., 2013). Accordingly, this study proposes the following hypotheses:

(1) H4: Social environment has an influencing on sport TID.
(2) H4a: Policy and system guarantee positively affect sport TID.
(3) H4b: The degree of emphasis on talent cultivation positively affects sport TID.
(4) H4c: School sports atmosphere positively affects sport TID.

## Methods

### Participants and Procedures

A total of 330 questionnaires were sent and collected. Invalid questionnaires were removed, and the returned questionnaires were carefully reviewed. Finally, 310 questionnaires were determined to be valid, with a questionnaire effective rate of 93.9%.

The information about the respondents in the confirmed valid questionnaire is as follows: (1) Gender and age composition: The female proportion was 48.38%, and the proportion of people under 30 years is 21.3% and that between 30 and 45 years is 36.7%; (2) The situation of professional title: In this survey, the title of professor accounts for 7%, and the title of associate professor was 9%. (3) Occupational distribution: The percentage of PE teachers was 30.64%, coaches were 24.84%, and the rest were PE administration and education administration personnel. (4) Education level: The proportion of those with doctoral degrees was 17.42%. (5) Type of work unit and work experience: The number of respondents working in colleges and universities accounted for nearly half, 48.39%. (6) Work experience: 13.2% had <3 years of experience, 27.9% had 3–5 years, 40.6% had 5–10 years, and 18.3% had more than 10 years of work experience.

The procedure is to distribute the questionnaires online (e.g., e-mail and Google online questionnaire) and offline and collect them after they are filled in. After repeatedly revising and determining the questionnaire, the researchers conducted a questionnaire survey on the factors affecting the sport TID in China's PE curriculum among PE teachers, coaches, and sports experts, and collected the sample data of the questionnaire survey.

The majority of the survey studies of performance relied on the questionnaire for date collection (Doherty, 1998). The respondents of this study are mainly concentrated in Guangdong province, according to the number of staff in the sports system in the Guangdong Provincial Statistical Yearbook 2021 (http://stats.gd.gov.cn/gdtjnj/), there were 1,874 sports coaches, 927 full-time teachers, and 5,224 administrative personnel. Overall, 330 participants were selected for this research. Then through the method of random sampling, an appropriate number of individuals are randomly selected in each subgroup. Last, based on the principle of stratified sampling, 77 people are selected from sports coaches, 38 teachers, and 215 sports management personnel. Hence, the sample size in this study is 330 sports system practitioners (i.e., administrative personnel, coaches, and school PE teachers). Sports experts, administrative personnel, middle school PE teachers, and coaches from Guangdong province were selected to represent the south of China. In this way, the questionnaire was distributed among the school PE and education system practitioners by random sampling.

### Measures

For the purpose of ensuring the validity of this questionnaire, it is designed from multiple angles. By reading the relevant literature and expert interviews, combined with the characteristics of school PE classes and talent growth environment, a questionnaire has been designed. After the questionnaire is collected, it is necessary to process information. The design of this questionnaire for this study was structured and multiple-choice type. It was divided into three parts. The first part comprised the basic information of the respondents, including personal information (gender, professional title, age, working unit, and education level) and basic information of the affiliation (affiliation category). These questions ensure the validity of the samples and allow the survey results to be classified and cross-analyzed. The second part is the evaluation of the influencing factors, which includes 12 influencing factors (Table 1), with the degree of importance ranging from high impact, moderate impact, low impact, rare impact to no impact, and 5, 4, 3, 2, and 1 points are given, respectively. The third part comprises other factors affecting the detection of sports talents in the school PE curriculum. This part of special measurement is mainly to collect some specific factors of the sports TID in school PE classes in China of this questionnaire. The aim of the part is to collect respondents' judgments on the degree of influence between variables.

**Table 1:** Measurement items.

**Number**	**Latent variable**	**Observation variable**
A1	Physical	Height and weight
A2		Motor ability index
A3		The quality of anthropometric index
B1	Psychological	The motivation of students to participate in sports
B2		Personal qualities
B3		Students' cognition of sports
C1	Coach	Professional level
C2		The relationship between coaches and students
C3		Moral education and management ability of coaches
D1	Environmental	Policy and system guarantee within the school
D2		Degree of emphasis on talent cultivation
D3		School sports atmosphere

### Statistics Analysis

**Figure 1:**
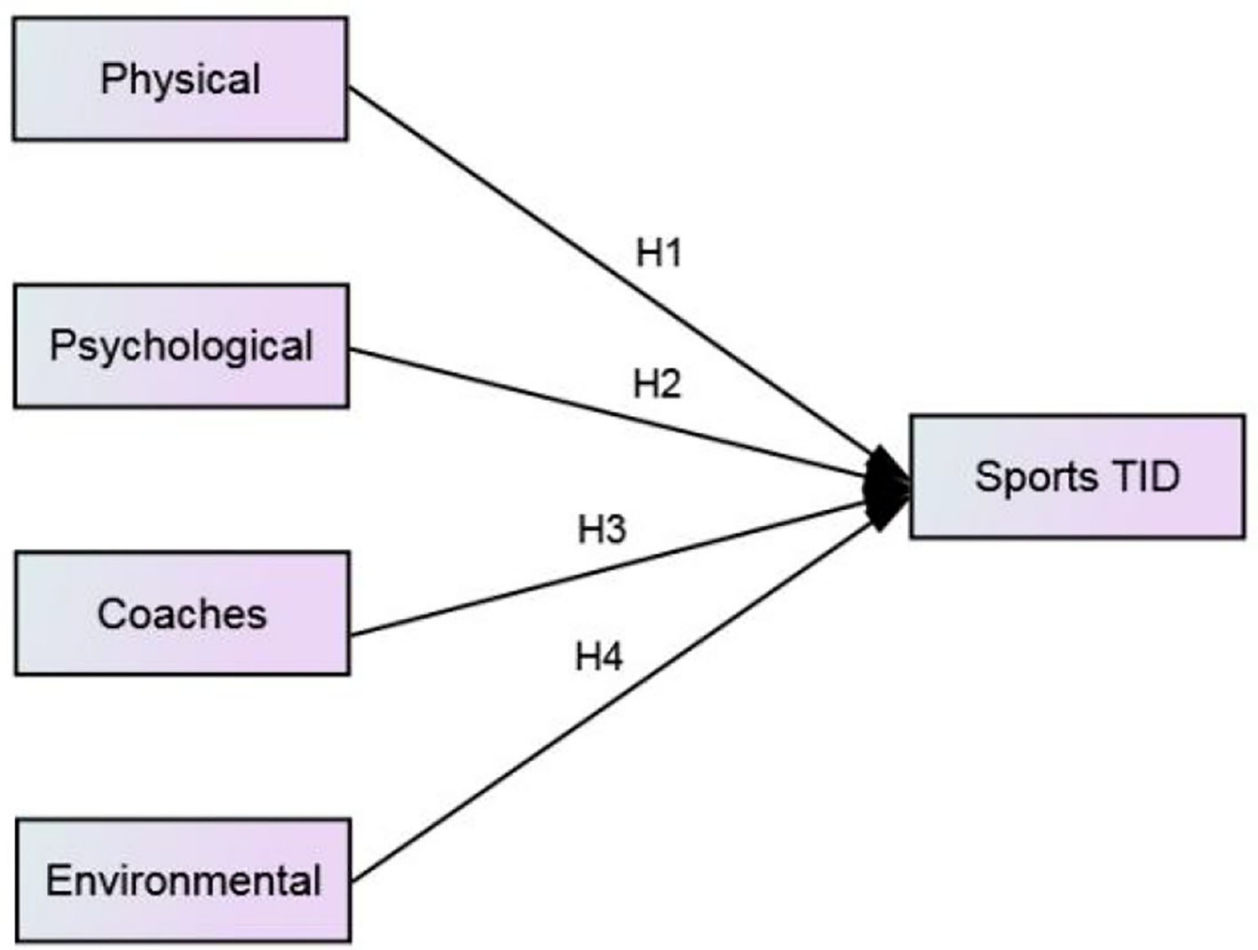
Theoretical model.

**Figure 2:**
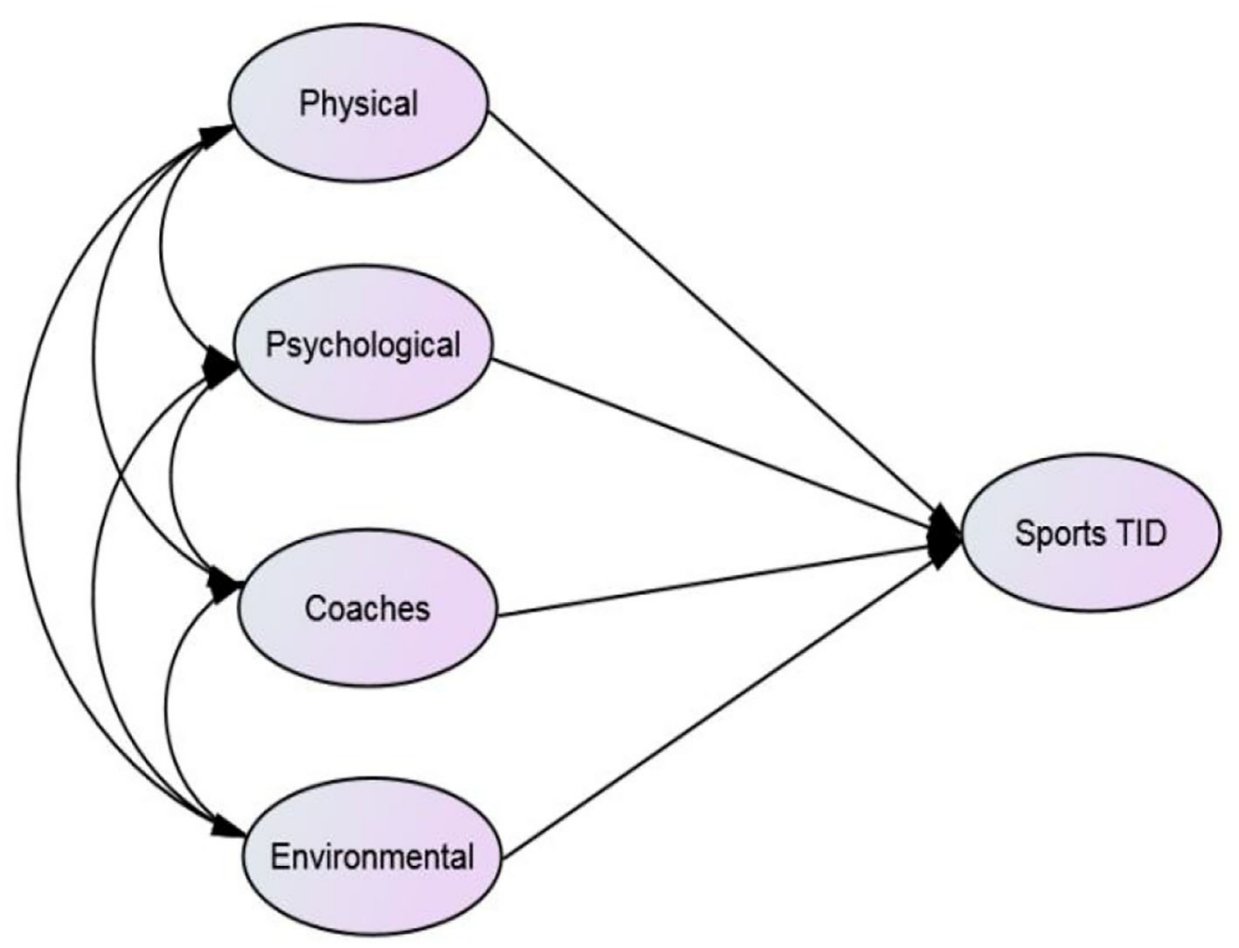
Conceptual model.

The structural equation model (SEM) is an empirical analysis model method that uses the linear equation system to express the relationship between observation variables and latent variables, as well as the relationship between latent variables. Compared with the traditional linear regression model, the SEM has the advantage of processing multiple variables at a time, allowing latent variables have multiple index variables and resulting in the reliability and validity of index variables, so that the model can adapt to a wider range and be more elastic. In recent years, the SEM has become the main analytical method in many fields such as sports (Marsh, 2007; Rocha and Chelladurai, 2012), education (Violato and Hecker, 2007; Khine, 2013; Green, 2016), and general management (Williams et al., 2009). The measurement model describes the relationship between latent variables and observed variables, while the structural model describes the relationship between latent variables (Figures 1, 2).

Analysis of moment structure (AMOS) can verify various measurement models and different path analysis models. In addition, multi-group analysis and the average structure number test can also be conducted. AMOS has four advantages (Cunningham and Wang, 2005): a graphical interface that helps see the changes that occur in the model and to establish relationships between the surfaces, easy to interpret and understand the meaning of the model diagrams created, a clear and intuitive way to read the results, and a full graphical display of the AMOS results.

In this research, the applicability of the four dimensions of TID was tested by SPSS26.0 according to the principal component reduction data set dimension, in which the KMO test value was 0.902, and the significance probability of the χ^2^ statistical value of the Bartlett sphere test was 0.000 (Table 2), indicating that the feasibility standard of principal component analysis was fully met.

**Table 2:** KMO and Bartlett sphere test.

**Kaiser-Meyer-Olkin measure of sampling adequacy**		**0.902**
Bartlett's test of sphericity	Approx. chi-square	1,366.274
	df	66
	Sig.	0.000

## Results

Each SEM has many latent variables, and the attributes of latent variables should meet the requirements of both validity and reliability (Raykov and Marcoulides, 2006). In order to ensure the suitability, validity, and reliability of the model, SPSS software was used for statistical analysis of the collected questionnaire data. If *p* ≤ 0.05, it means that the overall model is not significant, indicating that the model is consistent with the sample data. Otherwise, if *p* > 0.05, it is significant, indicating that the model is inconsistent with the sample data. After meeting the aforementioned requirements, the hypothesis test and modification of the model are conducted.

Based on the widely used criterion of Cronbach coefficient >0.7, the reliability of this model was tested on the basis of Churchill's (1979) criterion that the overall correlation coefficient of the item should not be <0.5. As shown in Table 3, Cronbach's α was 0.7, the composite reliability (C.R.) was higher than 0.7, and the average variance extracted (AVE) was also more than 0.5, except for the psychological AVE is less 0.5 (but above a reasonable range of 0.45). In this way, the model has good convergent validity.

**Table 3:** Reliability analysis.

**Latent** **variables**	**Observed** **variables**	**Standardized** **factor loading**	**Cronbach's** **alpha**	**C.R**.	**AVE**
Physical	A1	0.832	0.716	0.752	0.507
	A2	0.604			
	A3	0.680			
Psychological	B1	0.674	0.723	0.725	0.475
	B2	0.836			
	B3	0.590			
Coaches	C1	0.815	0.734	0.849	0.652
	C2	0.842			
	C3	0.764			
Environmental	D1	0.567	0.751	0.819	0.614
	D2	0.821			
	D3	0.722			

### Confirmatory Factor Analysis

Confirmatory factor analysis (CFA) is a part of SEM analysis and plays a significant role in SEM analysis (MacCallum and Austin, 2000; Brown, 2006). CFA mainly presents the commonality of factor load and measurement model factor variables in tables. The contents should include standardized and non-standardized load, standard error, significance, composition reliability, extraction of mean variance, and appropriate fitting indicators. Thus, CFA can provide details about the setting and evaluation of SEM for this study and increase confidence in the research results.

### Establishment of SEM

**Figure 3:**
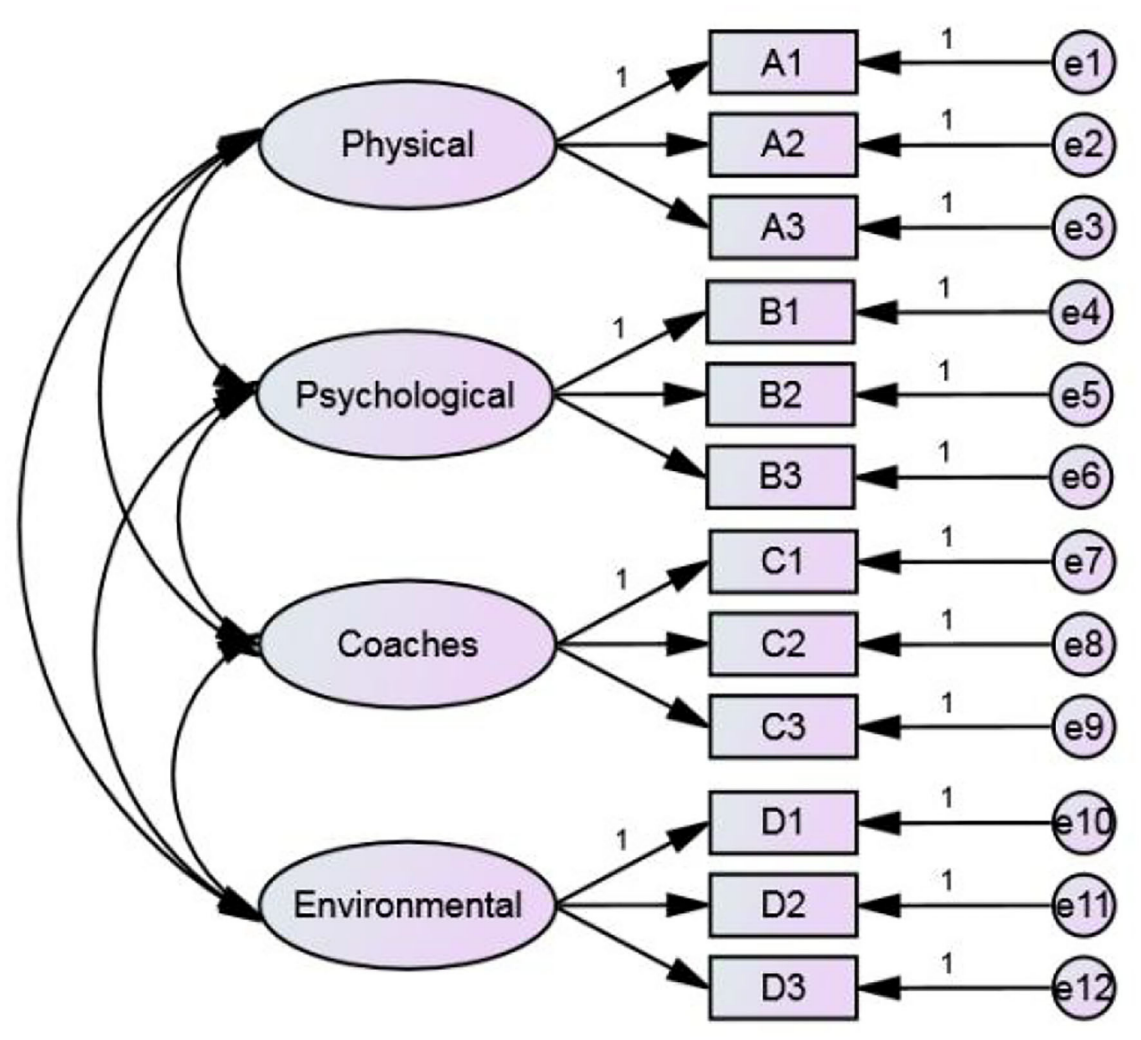
Confirmatory factor analysis model.

In this hypothetical model, there are four factors, and each factor has three observed variables and a total of 12 observed variables. All factors are the cause of the observed variable, so they point to the observed variable. When the observed variable is estimated by latent variable, there are two parts: explainable variance (variation) and unexplainable variance (variation). The square of the standardized factor load is the explainable variation, and there are 12 errors (unexplained variation) from e_1_ to e_12_. Meanwhile, there is a correlation between latent variables, so there are six covariances (correlation). The four-factor hypothetical model of sport TID is shown in Figure 3, which includes four facets and a total of 12 measurement indicators. All latent variables are exogenous variables. Exogenous variables affect endogenous variables (measurement indicators), and there is a complete correlation between latent variables. For this reason, the sport TID model has 12 endogenous variables and 16 exogenous variables (including 12 measurement errors and four exogenous latent variables). Because the four dimensions of sport TID are our hypothetical structure and do not exist in the data, in order to measure the hypothetical structure, the factor load of the first question of each structure is set to 1, and its variance is mapped to each facet of sport TID.

**Figure 4:**
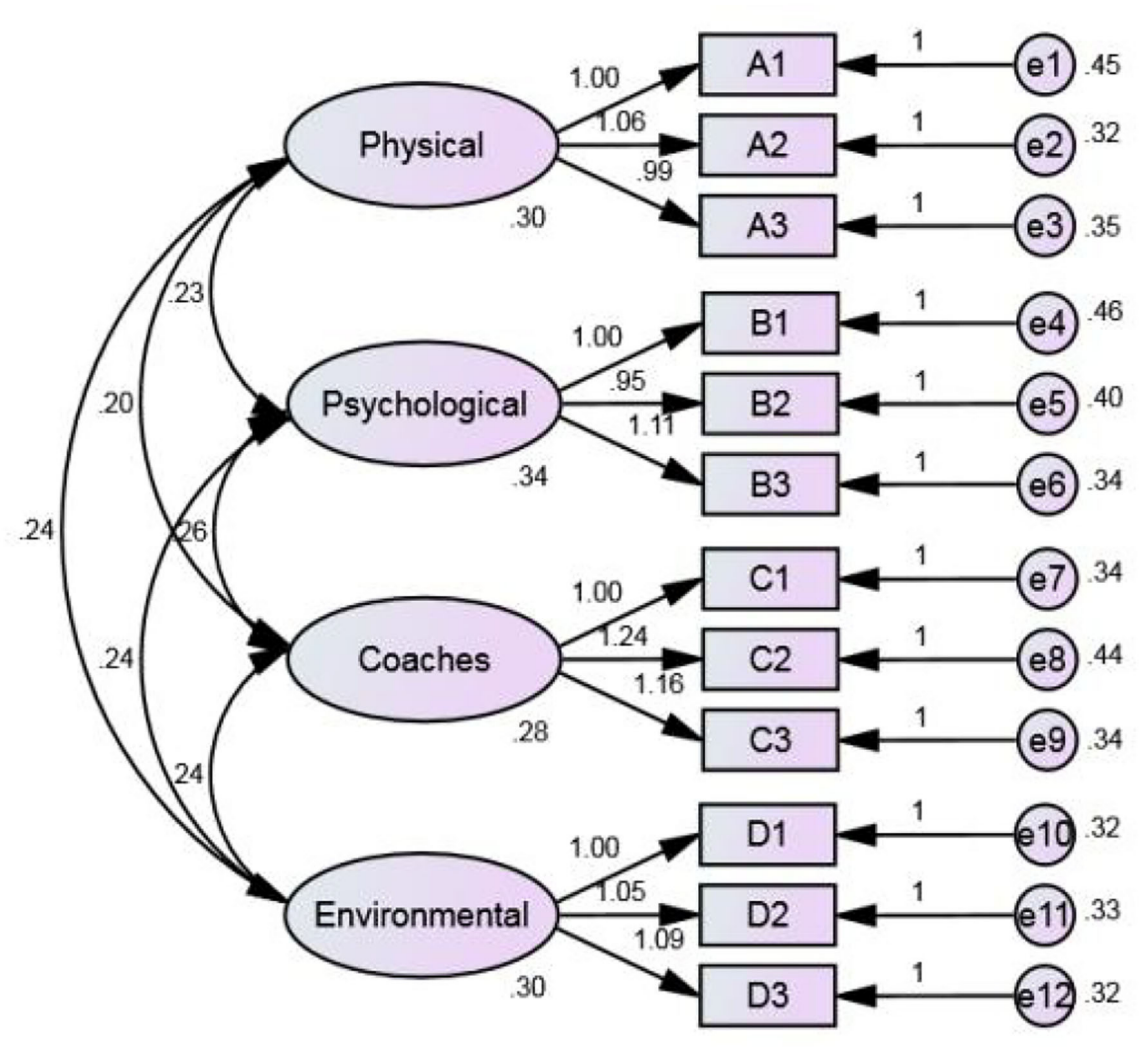
Unstandardized estimates.

**Figure 5:**
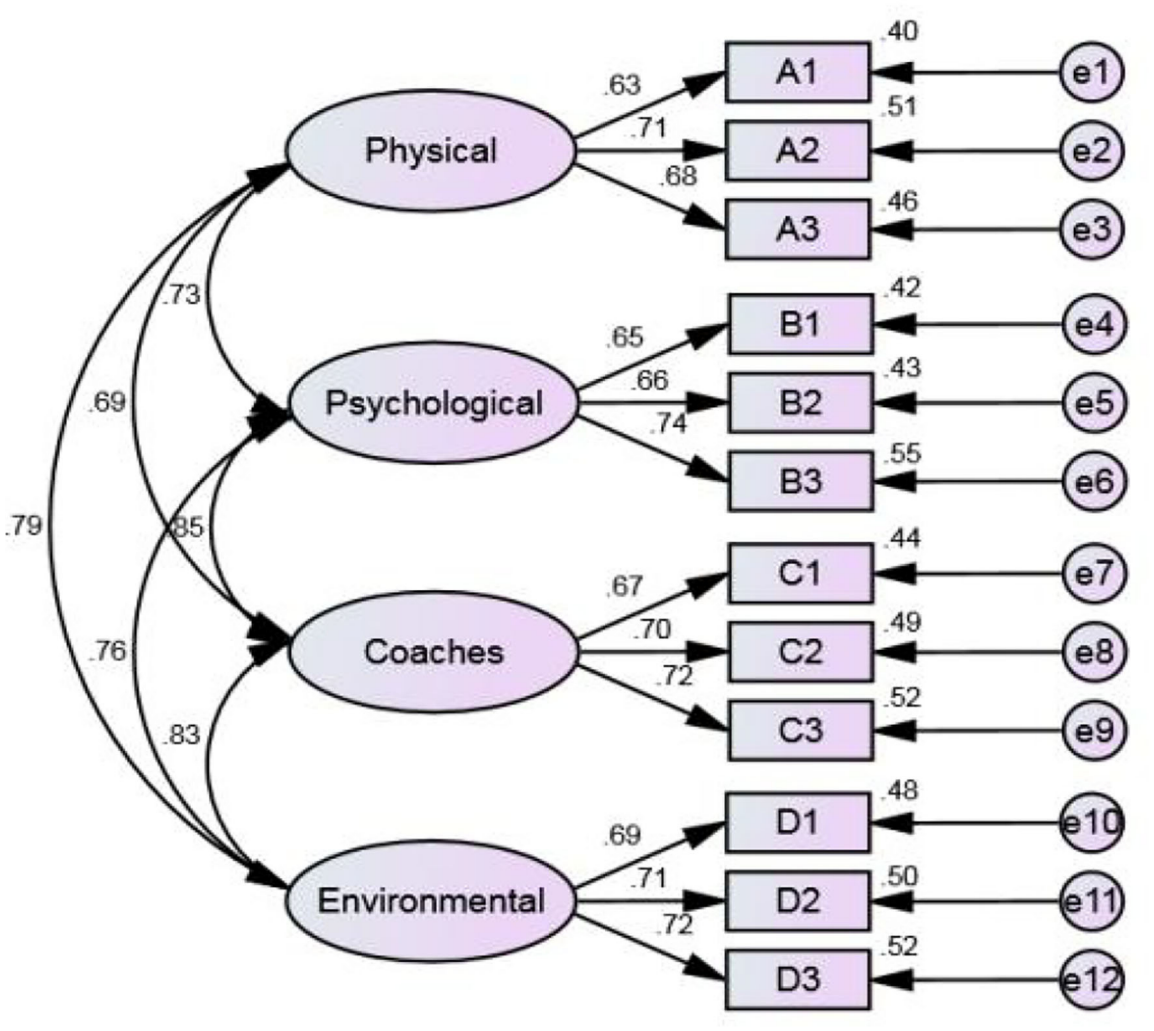
Standardized estimates.

After this study determines that the model can be estimated, the next step is to estimate the model parameters. The model in this research is analyzed by the maximum likelihood estimates (MLEs), which make the estimated results of all parameter values have the highest similarity with the actual data, and the MLE is completed by the iterative program. Table 3 shows the correlations matrix of all analyzed variables for TID, with three decimal places retained (Hoyle and Panter, 1995), for a total of 310 samples. Figure 4 shows the non-standardized estimated results and Figure 5 shows the standardized estimated results of CFA for sport TID.

### Model Test and Analysis

Table 4 lists the main fit indexes obtained from the structural model test in detail. After comparing with the given recommended values of the fitness indicators, the indices of the fit indicators fall within the recommended values, except for the AGFI value, which is just above the recommended value of 0.9. It can be seen that the setting of the theoretical model in this study is acceptable.

**Table 4:** Sample correlations (group number 1).

	**A1**	**A2**	**A3**	**B1**	**B2**	**B3**	**C1**	**C2**	**C3**	**D1**	**D2**	**D3**
A1	1.000											
A2	0.451	1.000										
A3	0.456	0.466	1.000									
B1	0.353	0.367	0.344	1.000								
B2	0.286	0.315	0.380	0.441	1.000							
B3	0.303	0.344	0.374	0.468	0.487	1.000						
C1	0.348	0.445	0.387	0.329	0.466	0.414	1.000					
C2	0.345	0.306	0.287	0.359	0.333	0.505	0.389	1.000				
C3	0.257	0.317	0.279	0.430	0.353	0.446	0.447	0.601	1.000			
D1	0.357	0.353	0.418	0.352	0.386	0.410	0.487	0.425	0.401	1.000		
D2	0.302	0.481	0.330	0.316	0.343	0.430	0.480	0.314	0.388	0.466	1.000	
D3	0.317	0.449	0.356	0.339	0.338	0.402	0.413	0.428	0.396	0.481	0.556	1.000

Meanwhile, Table 5 shows the parameter estimates after model testing, including unstandardized estimates, standard errors, significance, and standardized estimates. As shown in Table 5, all estimated parameters are significant with standardized estimates ranging from 0.63 to 0.74 and squared multiple correlations (SMC) ranging from 0.39 to 0.54, in accordance with the requirements suggested by Fornell and Larcker (1981).

**Table 5:** Fit index value of SEM.

**Fit indices**	**Recommended value**	**Fitting value**
χ^2^	The smaller, the better	118.033
χ^2^/df	<3.0	2.495
GFI	>0.9	0.939
AGFI	>0.9	0.901
RMSEA	<0.08	0.069
NNFI	>0.9	0.915
IFI	>0.9	0.948
CFI	>0.9	0.947

Totally, four factors were selected by principal component analysis in SPSS, as shown in Table 6. A1, A2, and A3 were clustered as one factor and named physical factor. B1, B2, and B3 were clustered as one factor and named psychological factor. C1, C2, and C3 were clustered as one factor and named coaches factor. D1, D2, and D3 were clustered as one factor and named environmental factor. In the meantime, Table 7 shows the interpretation of the total variance interpretation after rotation. Among them, the cumulative variance contribution rate of the rotation of the first four factors reaches 67.16% (Table 8). In general social investigation and research, the contribution rate of more than 60% is also in line with the requirements, which can better explain that these factors can represent the main content and information.

**Table 6:** CFA parameter estimation results.

**Paths**	**Unstandardized** **estimate**	**S.E**.	***T*-value *p***	**Standardized** **estimate**	**SMC** **(*R*^2^)**
A1 < – Physical	1.000			0.632	0.399
A2 < – Physical	1.060	0.115	9.258***	0.715	0.511
A3 < – Physical	0.992	0.110	8.988***	0.679	0.461
B1 < – Psychological	1.000			0.648	0.420
B2 < – Psychological	0.951	0.102	9.290***	0.657	0.431
B3 < – Psychological	1.112	0.110	10.089***	0.740	0.548
C1 < – Coaches	1.000			0.667	0.445
C2 < – Coaches	1.242	0.122	10.175***	0.703	0.494
C3 < – Coaches	1.158	0.111	10.393***	0.723	0.523
D1 < – Environmental	1.000			0.694	0.481
D2 < – Environmental	1.054	0.099	10.613***	0.709	0.502
D3 < – Environmental	1.090	0.101	10.791***	0.724	0.524

***p-value is < 0.001.

**Table 7:** Rotating component matrix.

**Items**	**Factor loading values**				**Factor**
	**1**	**2**	**3**	**4**	
A1	0.832				Physical
A2	0.604				
A3	0.680				
B1		0.614			Psychological
B2		0.836			
B3		0.590			
C1			0.815		Coaches
C2			0.842		
C3			0.764		
D1				0.567	Environmental
D2				0.821	
D3				0.722	

**Table 8:** Total variance interpretation.

**Component**	**Initial eigenvalue**			**Rotating the sum of squares and loads**		
	**Total**	**% of variance**	**Cumulation %**	**Total**	**% of variance**	**Cumulation %**
1	5.311	44.260	44.260	2.459	20.490	20.490
2	1.062	8.849	53.110	1.891	15.758	36.248
3	0.904	7.533	60.643	1.867	15.559	51.807
4	0.782	6.521	67.164	1.843	15.356	67.164
5	0.671	5.595	72.758			
6	0.601	5.012	77.771			
7	0.548	4.570	82.341			
8	0.523	4.355	86.696			
9	0.476	3.967	90.663			
10	0.418	3.487	94.150			
11	0.389	3.241	97.391			
12	0.313	2.609	100.000			

*Extraction method: principal component analysis*.

The aforementioned data and models showed that the path coefficient is significant. Physical, psychological, coach, and environmental factors are the four main aspects that affect the sport TID in the PE curriculum in China.

## Discussion

In this study, the unstandardized parameter estimates in Figure 4 and the standardized parameter estimates in Figure 5 were obtained by AMOS of the hypothesis model. Based on the previous reliability and validity analysis and the validation factor analysis, the model of factors affecting the sport TID was tested and revised in this way. Since the sport TID model is not bad overall, the model does not need any correction. So, from the sport TID model, we can know that the main influencing factors are physical, mental, coaching staff, and environment.

Physical factor has an effect on sport TID, namely, H1 is correct. Physical factors include height and weight, motor ability, and anthropometric indicators. The proportion of motor ability in factor load is the largest, which shows that for the identification of sports talents, students' motor ability is a major influencing factor to find potential athletes, so more attention should be paid to motor ability in the sport TID in future. In addition, students' height and weight, as well as anthropometric index, also play an important and indispensable role in sport TID.

Psychological factor has an impact on sport TID, namely, H2 is correct. Psychological factors include sport motivation, personal quality, and students' cognition of sports. The factor coefficient of personal quality is the largest. This shows that for the sport TID, students' personal quality is crucial, and only a good psychological quality can endure a certain amount of physical training load. Second, students' motivation and cognition for sports can also effectively identify sports talents. Some sports psychology studies still show that sport motivation is an important factor affecting sport TID (Abbott and Collins, 2004; Höner and Feichtinger, 2016).

The coach factor has an effect on sport TID, namely, H3 is correct. The factors of coaches mainly include professional and skill levels, relationship with students, and management ability. As an important medium for cultivating and discovering sports talents, coaches are both organizers and instructors, who play a powerful role in promoting the identification and development of sports talents. Therefore, in the process of sport TID, the factors related to coaches also need to be concerned.

The environmental factor has an impact on sport TID, namely, H4 is correct. The environmental factors in this study are mainly at the social level, including policy and systemic guarantees, attention to the sport TID, and the school sport atmosphere. The social environment (e.g., family characteristics and children's growth environment) is relatively a complex factor because the times are constantly updating and changing, which also has a great impact on the sport TID (Bailey and Morley, 2006). Sport TID is for the society demands and for the development of sports. From this aspect, environmental factors are closely related to the sport TID to a large extent. As mentioned before, we must grasp the environmental factors, make good use of social policies, and improve the work of sport TID.

### Implication

#### Comprehensively Develop Students' Physical Quality

The physical qualities of students generally include strength, speed, endurance, agility, and flexibility. Good physical quality is the basis for mastering sports skills and tactics as well as improving sports performance. The more comprehensive the physical quality, the more conducive to the mastery of sports skills and tactics. The physical quality development of primary and middle school students has its own characteristics, including growth and sensitive period; meanwhile, there is an imbalance in the development of various qualities in different age stages. Thus, when identifying sports talent in the PE curriculum, we should pay attention to the characteristics and sensitive period of students' physical quality development, as well as adopt different physical quality testing methods to identify sports talents in line with the specific conditions of students' age and grade. In addition, anthropometric indicators are also related to students' growth and development, and it is necessary to notice the changes of students' body shape, which are wavy, phased, gender differences, and imbalance. In this way, good use of the important role of anthropometry in the sport TID must be highlighted. With the application of anthropometric data and comprehensively considering the relationship among human body's morphological structure, physiology, physical quality, and talent identification technology, we can improve the success rate of youth sport TID and reduce the missed selection rate.

#### Grasp the Psychological Status of Students

The psychological state of students is also closely related to the sport TID. The psychological basis of athletes includes two aspects: individual psychological characteristics and sports psychological process. The most vital personality psychological characteristics that determine the traits of students' sports behavior are sport motivation (Clancy et al., 2016), interest in training and competition, cognition of sports, personal personality, and temperament (Bailey and Morley, 2006; Li et al., 2021). The physiological basis of temperament depends on the types of high-level neural activities, which are generally divided into four types: excitatory, lively, quiet, and depression (Wytykowska et al., 2022). These four neural types have different characteristics in people's behavior. Generally speaking, different sports items have different requirements for individual psychological characteristics (Gimeno et al., 2007). However, on the whole, a student who can bear hardships and love sports in his/her heart will certainly have a good psychological performance. At the same time, some students' good psychological performance is innate and affected by genetic factors, but it will also be affected by the acquired influence of society, family, and school education. Therefore, students' psychological state is closely related to the sport TID.

### Improve the Professional Quality of Coaches

As an important organizer and executor of sport TID, coaches play an inseparable role in the process of sport TID. It is always said that swift horses are usually found but not the same as the person who has good judgments to spot them. A professional and experienced coach can fully understand and tap a student's sports potential, as well as can predict whether a student has the ability to become an excellent athlete in future. The professional quality and ability of coaches have laid a theoretical and practical foundation for them to identify sports talents. At the same time, the more familiar coaches are with students, the more they can understand students' inner world and whether students truly like sports, so as to better know which students have the qualification to become athletes. Additionally, there is a certain relationship between coaches' management consciousness and sport TID. A coach with strong management consciousness and organizational ability can have good control over students and discover, cultivate, and select talents for the country.

### Make Good Use of Social Environment and Policies

Under the background of the policy of “Sports Power” and “Talents Rejuvenate the Country,” policy plays a critical role in promoting the work of school PE and TID. In particular, the reform of the school PE curriculum and emphasis on the training of sports talents have been greatly promoted, which will further accelerate the identification of sports talents. A strong campus sports atmosphere can stimulate more students to participate in sports; with more participation base the more talent can be identified. Hence, for sport TID, the overall external environment is also very important, and it will affect school PE development, talent training, and guarantee from all aspects, which is inseparable with TID. In this way, with the implementation and guidance of the policy, the identification of school sports talents will be more favorable to conduct.

## Conclusion

With the development of big data, artificial intelligence, and information technologies, sports talents may be more effectively and accurately identified in future, making the identification of sports talents more scientific and effective. Through big data analysis, we can make a comprehensive evaluation on the learning environment, training conditions, physical fitness, competition results, team cooperation, and other indicators of talent growth in each stage; make a scientific evaluation on the development potential of sports talents; and then predict the growth and development of talents. Therefore, we can better identify the types and characteristics of each sports talent and provide corresponding training as well as guidance, which will also be conducive to the growth and development of sports talents, and the most important thing is to ensure the sustainable development of sports. This research analyzed the factors affecting the sport TID in the PE curriculum through the CFA in the SEM. Through the analysis of data and model, it is known that the main factors affecting the identification of sports talents in the PE curriculum include four aspects: physical, psychological, coaches, and environment.

Nevertheless, this study is not without limitations. Because all kinds of schools at all levels have different management systems and processing methods in the detection, cultivation, selection, after-school sports training, sports development, and other aspects of their own sports talents, we should also consider the differences of schools. Except that, in the actual school PE work, there are still other factors affecting the identification of sports talents, such as the level of regional economic development, the investment of school funds, and traditional sports culture. Therefore, the model analysis of this study does not cover all the interfering factors. Consequently, further research need to select more theoretical models and influencing factors, to construct a more comprehensive theoretical framework and better analyze the influencing factors of TID in the school PE curriculum.

## Data Availability Statement

The original contributions presented in the study are included in the article/supplementary material, further inquiries can be directed to the corresponding author/s.

## Author Contributions

CX and HL: conceptualization. CX: methodology, software, resources, writing—original draft preparation, and visualization. CX and TT: validation. CX and NI: formal analysis. HL: investigation, project administration, and funding acquisition. CX, HL, and TT: data curation. HL and TT: writing—review and editing and supervision. All authors have read and agreed to the published version of the manuscript.

## Funding

The authors acknowledge financial support from the Guangzhou Education Science Planning 2020 Youth Special Project (202012684) and UPM Journal Publication Fund.

## Conflict of Interest

The authors declare that the research was conducted in the absence of any commercial or financial relationships that could be construed as a potential conflict of interest.

## Publisher's Note

All claims expressed in this article are solely those of the authors and do not necessarily represent those of their affiliated organizations, or those of the publisher, the editors and the reviewers. Any product that may be evaluated in this article, or claim that may be made by its manufacturer, is not guaranteed or endorsed by the publisher.
